# Retinal and Choroidal Effects of Continuous Positive Airway Pressure as Treatment for Sleep Apnea: Results at 12 Months

**DOI:** 10.3390/ijerph191912637

**Published:** 2022-10-03

**Authors:** Pedro Naranjo-Bonilla, Rafael Giménez-Gómez, María del Carmen Muñoz-Villanueva, Bernabé Jurado-Gámez

**Affiliations:** 1 Ophthalmology Department, Maimonides Biomedical Research Institute of Cordoba (IMIBIC), 14004 Córdoba, Spain; 2 Ophthalmology Department, University Hospital Juan Ramón Jiménez, 21005 Huelva, Spain; 3 Ophthalmology Department, Maimonides Biomedical Research Institute of Córdoba (IMIBIC), University Hospital Reina Sofía, 14004 Córdoba, Spain; 4 Primary Care Health Centre, Córdoba-Guadalquivir Health District, 14011 Córdoba, Spain; 5 Respiratory Department, Maimonides Biomedical Research Institute of Cordoba (IMIBIC), University Hospital Reina Sofía, University of Córdoba, 14004 Córdoba, Spain

**Keywords:** obstructive sleep apnea, CPAP, optical coherence tomography, retinal nerve fiber layer, retinal ganglion cells, inner plexiform layer, choroid, photoreceptor layer

## Abstract

Background: To determine the impacts of continuous positive airway pressure (CPAP) treatment on retinal and choroidal thickness measurement in individuals with obstructive sleep apnea (OSA). Methods: Participants were 28 patients with OSA treated with CPAP who were enrolled immediately after diagnosis and graded according to the apnea hypopnea index (AHI) determined in an overnight polysomnography. Inclusion criteria were a new diagnosis of OSA and an indication for CPAP. Participants underwent a full ophthalmologic examination including standard automated perimetry (SAP) and optical coherence tomography (OCT) at the levels peripapillary, macular, and choroidal before CPAP onset, and after three and twelve months of CPAP. The data compared before and after treatment were intraocular pressure, SAP, and the thicknesses peripapillary retinal nerve fiber layer (pRNFL), total retinal (TR), retinal ganglion cell layer (RGCL), inner plexiform layer (IPL), photoreceptor layer (PL), and choroidal. Results: After 3 months of CPAP, we observed thickening of the pRNFL (in 5/6 subfields) (*p* < 0.004) and TR (in 5/9 subfields) (*p* < 0.010). At 12 months, thickening persisted in these layers, this time affecting 2/6 and 2/9 subfields, respectively (*p* < 0.012 and *p* < 0.001, respectively). Choroidal thinning was observed at the temporal level at both 3 and 12 months compared to measurements before starting CPAP treatment (*p* = 0.014 and *p* = 0.038, respectively). SAP remained unchanged. Intraocular pressure was higher at 12 months than at 3 months (*p* = 0.001). Conclusions: 12 months of CPAP avoids retinal thinning and normalizes choroidal thickness in OSA patients.

## 1. Introduction

Obstructive sleep apnea (OSA) is a chronic respiratory disorder in which the upper airway completely (apnea) or partially (hypopnea) collapses during sleep [1]. Its prevalence in the general population is 5–15% [2]. Individuals with moderate or severe OSA have a greater risk of experiencing a cardiovascular event [3], and also show a higher all-cause mortality [4]. Sleep apnea has also been associated with ocular diseases [5] such as glaucoma [6,7,8,9,10], which has been attributed to a reduced capacity to regulate ocular blood flow [11,12].

Retinal blood vessels have the capacity of autoregulation [11], essentially depending on blood oxygen levels [13]. In conditions of hypoxia, oxidative stress at the level of the vascular endothelium leads to the inactivation of nitric oxide [11], and this suppresses vasodilation, thus promoting vasoconstriction through increased levels of endothelin-1 [14]. The vessels of the retina are found throughout its thickness, and in some zones, such as around the optic disk, they may occupy a thickness of 120 µm [15]. At this peripapillary level, the larger arterioles occur in the retinal nerve fiber layer (RNFL). Patients with moderate to severe OSA feature a peripapillary RNFL (pRNFL) thickness of 95–100 µm [16]. Hence, the vascular compartment seems to have a considerable impact on retinal thickness, especially in the peripapillary zone and in its RNFL. In effect, a thinner pRNFL has been observed in subjects with thinner vessels [17]. Moreover, OSA has been associated with atrophy of this layer [6,18]. Glaucomatous neuropathy is also characterized by progressive damage to the inner retinal layer, mostly affecting the RNFL and retinal ganglion cell layer (RGCL) [19].

The choroid shows a reduced capacity for autoregulation and is subjected to systemic changes [11]. In prior work, we detected a positive correlation between the apnea hypopnea index (AHI) and choroidal thickness at the foveal, nasal, and temporal levels [16]. This determined that subjects with more severe OSA had a thicker choroid.

Continuous positive airway pressure (CPAP) is the treatment of choice for individuals with moderate to severe OSA. This therapy has been proven to improve respiratory disorders and endothelial damage [20]. However, few studies have examined the effects of CPAP on the retina and choroid of subjects with OSA. This disease is complex and involves several systemic disorders produced as a consequence of inflammatory and vascular changes. Three months of CPAP therapy has been shown to preserve retinal thickness and reduce choroidal thickness in OSA patients [16]. However, the effects of CPAP therapy produced in the longer term once retinal inflammation and vascular changes are controlled remain unknown. In this study, we sought to assess changes produced in visual function and in the thicknesses pRNFL, total retinal (TR), RGCL, inner plexiform layer (IPL), photoreceptor layer (PL), and choroidal in subjects with OSA just before CPAP treatment onset and at follow-up visits scheduled for 3 and 12 months after treatment.

## 2. Methods

### 2.1. Study Participants

This was a cohort study with 12 months of prospective follow up. Subjects were consecutively enrolled when newly diagnosed with OSA, and when CPAP treatment was indicated [21] according to the results of an overnight polysomnography at the Sleep Unit of our center (Hospital Universitario Reina Sofia, Cordoba, Spain). The study protocol received approval from the hospital Ethics Committee (Act no. 219, Ref: 2240). Written informed consent was obtained from all participants.

Candidate participants were assessed at the Ophthalmology Dept. within three days of OSA diagnosis. After this baseline ophthalmology visit, participants were started on CPAP. Those who fulfilled the selection criteria were scheduled for a second and a third visit at 3 months and 12 months. One ophthalmologist (PNB) conducted all assessments without knowing whether the subject’s visit was baseline, 3, or 12 months after CPAP onset. Details of the overnight polysomnography, CPAP treatment, and follow-up ophthalmologic examinations may be found in our previous report [16].

As inclusion criteria, it was required that participants were older than 18 years and were diagnosed with OSA at the study outset, along with an indication for CPAP [21]. Ophthalmologic requirements were a visual acuity >0.6, sphere in the range of ±5 diopters, cylinder in the range of ±3 diopters, and corneal angle > III Shaffer. Participants were excluded if they had a systemic disease that could affect RNFL thickness or had eye surgery in the past 6 months or if they had any of the following conditions: previous ocular trauma, opaque ocular media, uveitis, retinal disease, optic neuritis, or glaucoma.

### 2.2. Polysomnography

The measurements made in the overnight polysomnography were apnea hypopnea AHI or total number of apneas and hypopneas per hour of sleep, minimum peripheral oxygen saturation (SpO_2_), oxygen desaturation index > 3% defined as the number of SpO_2_ decreases ≥ 3% per hour, and the percentage sleep time spent at SpO_2_ ≤ 90% (T90). OSA severity was classified according to AHI [21] as mild (AHI ≥ 5 and < 15 events/h), moderate (AHI ≥ 15 and <30 events/h), or severe (AHI ≥ 30 events/h).

### 2.3. Ophthalmologic Examination

The measurements made in each visit were: best-corrected visual acuity, slit lamp exam, intraocular pressure (IOP) through applanation tonometry (mean of 3 measures), gonioscopy and fundus exam, and standard automated perimetry (SAP) through Swedish Interactive Threshold Algorithm standard 30-2 Humphrey automated perimetry (Carl Zeiss Meditec Inc., Dublin, CA, USA). For pRNFL, TR, RGCL, IPL, PL, and choroidal thickness measurements, we used the real-time eye tracking system of the Spectralis spectral domain optical coherence tomography (SD-OCT) device (software version 5.4.7.0) after pupil dilation.

### 2.4. CPAP

After the baseline ophthalmologic exam, subjects accepting treatment were started on CPAP therapy. Then, 3 and 12 months later, the treatment adherence information recorded by the device’s software was obtained. Adequate adherence to CPAP was defined as its use for >3.5 h per night [21,22].

### 2.5. Statistical Analysis

Qualitative variables are provided as counts (n) and proportions (%), and quantitative variables as their means and standard deviations, medians and interquartile ranges, and minimum and maximum values. The Shapiro–Wilk test was used to check the normal distribution of data and the Levene test to confirm the homogeneity of variances. Parametric tests were used to compare normally distributed data and the corresponding non-parametric test was used if this was not the case.

Data were compared by repeated measures ANOVA (parametric) and the Friedman test (non parametric). As post-hoc tests for multiple comparisons, we used Bonferroni correction and the Wilcoxon test (according to the normality of the data distribution). To examine intraobserver reproducibility in the choroid analysis, we randomly selected 20 scans at the levels macular, temporal, and nasal and calculated intraclass correlation coefficients (ICC).

Correlation between polysomnographic variables before CPAP treatment and changes in ophthalmologic variables after CPAP treatment in OSA patients were assessed through Spearman’s rank correlation.

All statistical tests were performed using the software package SPSS v.25 (IBM, Armonk, New York, NY, USA). Significance was set at *p* < 0.05. All comparisons were two-tailed.

## 3. Results

**Figure 1:**
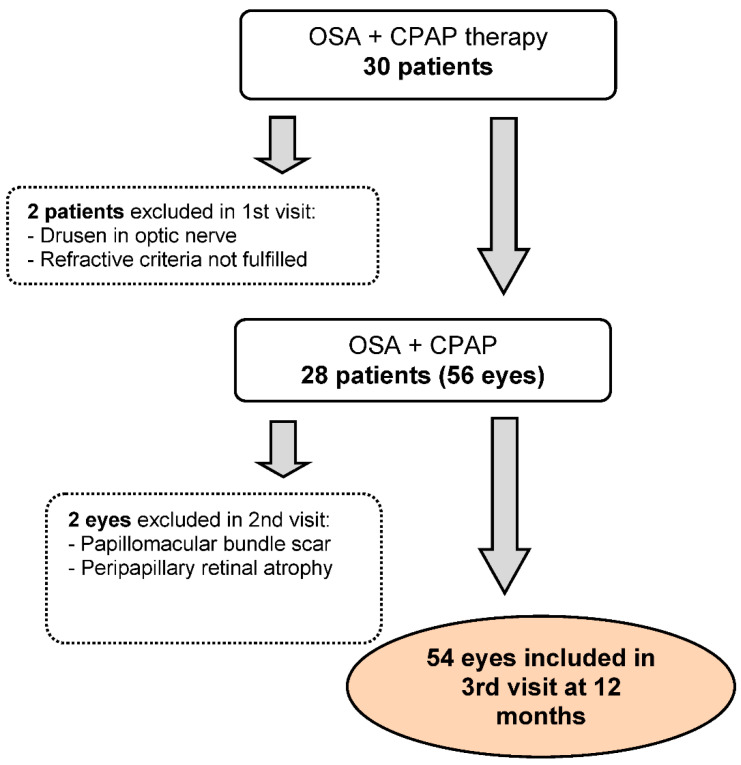
Enrolment of patients with obstructive sleep apnea (OSA) subjected to continuous positive airway pressure (CPAP) treatment.

Out of 30 patients initially enrolled, 54 eyes of 28 participants were finally included. The enrolment process is detailed in Figure 1.

Baseline patient characteristics are provided in Table 1. Most subjects had severe OSA and showed an AHI > 30 events/h. The mean number of hours per night of CPAP was adequate (5.09 ± 1.83) h. The prevalence of glaucoma was high (7.14%). No changes were observed in body mass index (*p* < 0.05) over the 12 months of follow up.

In Table 2, we provide the IOP, visual acuity, and SAP data obtained at baseline and in the 3- and 12-month follow-up visits. These data reveal an increase in IOP at 12 months of follow up compared to 3 months.

The changes observed in pRNFL thickness during the 12-month study period are provided in Table 3. At 3 months, this layer had significantly thickened at the levels global (G), temporal (T), superonasal (SN), nasal (N), and inferonasal (IN) compared to measurements made at baseline. In subfield G, thickness was greater at 12 months than before CPAP treatment was initiated. Negative correlation emerged between RNFL T subfield thickness and T90 at baseline (r = −0.338, *p* = 0.013), after 3 months (r = −0.334, *p* = 0.017) and after 12 months (r = −0.421, *p* = 0.002) of CPAP treatment, while positive correlation was detected between RNFL SN subfield thickness and T90 after 12 months of CPAP (r = +0.290, *p* = 0.033).

The TR, RGCL, IPL, and PL thickness data obtained at the macular level at baseline, 3 months, and 12 months are shown in Table 4. Greater variation was observed for the whole retina (TR), which was thicker at 3 months compared to baseline measurements in the subfields fovea (F), inferior inner macula (IIM), inferior outer macula (IOM), temporal outer macula (TOM), and superior inner macula (SIM). At the levels IIM and temporal inner macula (TIM), we observed a greater TR thickness at 12 months compared to baseline. No changes in RGCL emerged at 3 months although we did note thickening of this layer in the subfield SIM at 12 months compared to baseline or 3 months. Similar behavior was noted for IPL. Thus, we only observed its increased thickness in the TIM subfield at 3 months and in nasal inner macula (NIM) at 12 months. No modification in PL thickness was observed after 12 months of follow up.

In Table 5, we describe the changes in choroid thickness produced, namely thinning after the start of CPAP therapy at the temporal level both after 3 and 12 months of follow up. Nasal choroidal thickness and AHI were positively correlated at baseline (r = 0.359, *p* = 0.013), and after 3 months (r = 0.363, *p* = 0.015) and 12 months (r = 0.432, *p* = 0.002) of CPAP treatment.

Our analysis of intraobserver reproducibility of the choroid measurements returned ICCs of 0.94, 0.87, and 0.80 for 20 random scans at the levels macular, temporal, and nasal, respectively.

**Figure 2:**
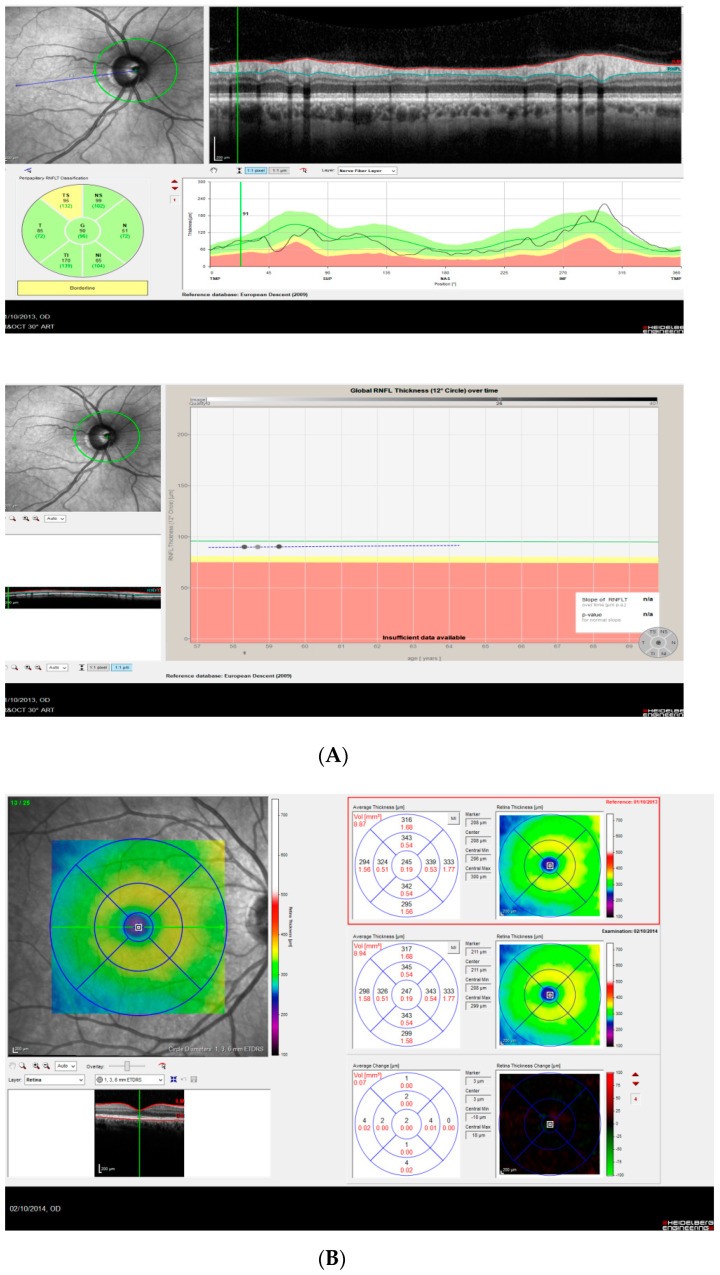

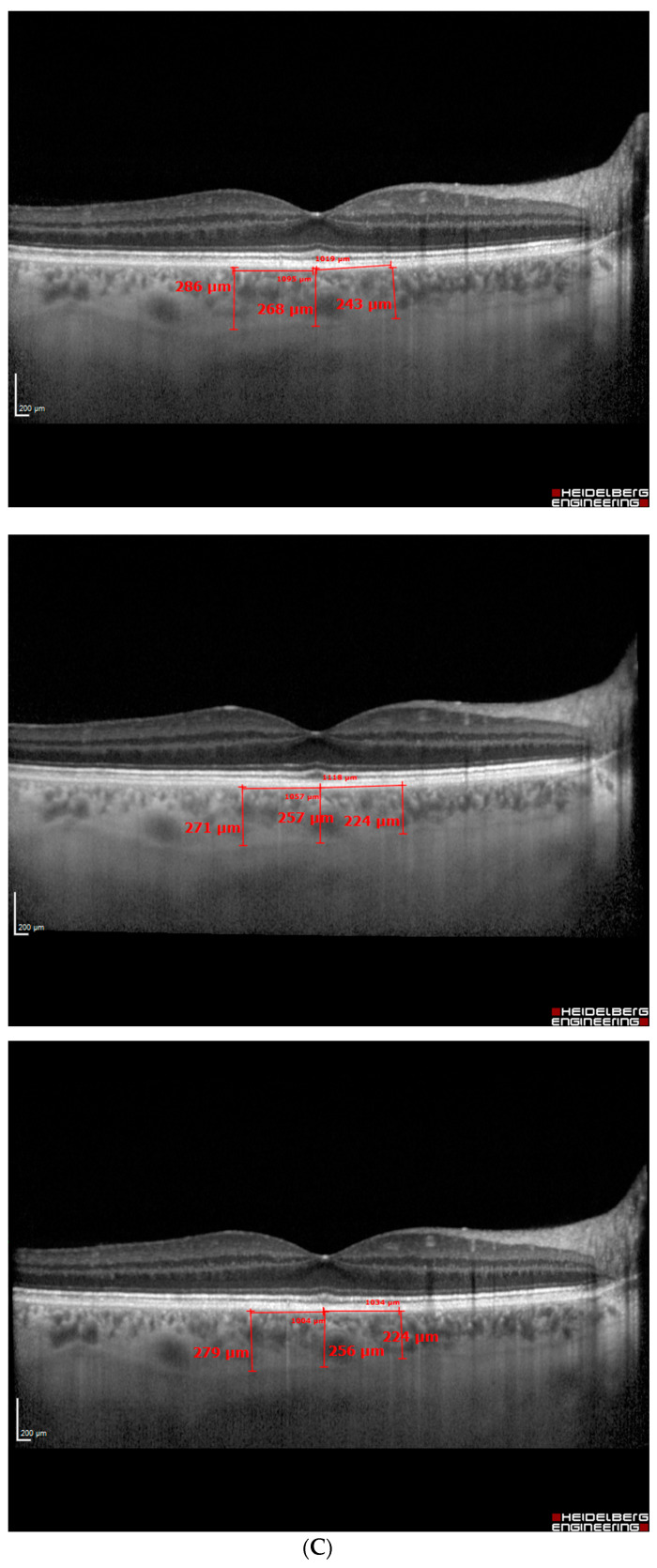
(**A**–**C**) A 54-year-old man with an apnea hypopnea index (AHI) of 54.8/h received nasal continuous positive airway pressure (CPAP) treatment starting 2 October 2013. Peripapillary retinal nerve fiber layer (pRNFL), total retinal (TR), and choroidal thickness measurements were taken on 1 October 2013 (baseline), on 14 January 2013 (after 3 months of CPAP) and on 2 October 2014 (after 12 months of CPAP). Following CPAP, AHI decreased to 6.1/h. (**A**) pRNFL thickness in the right eye (top: baseline, bottom: global RNFL thickness over 3 visits). Note the overall increasing trend of the curve. (**B**) TR thickness 12 months after CPAP treatment vs. baseline. Note the retinal thickening in the last EDTRS subfield. (**C**) Choroidal thickness in the right eye at the levels temporal, subfoveal, and nasal (top: baseline, middle: after 3 months of CPAP, bottom: after 12 months of CPAP).

As an example, we provide the OCT report obtained in a 58-year-old man at baseline, and after 3 and 12 months of CPAP therapy (Figure 2).

## 4. Discussion

In this study, we assessed visual function and the behavior of the retina and choroid in individuals with OSA before and after 3 and 12 months of CPAP therapy. We observed no changes in vision over the 12 months of treatment. In contrast, compared to before treatment onset, retinal thickness increased while choroidal thickness diminished at all follow-up times.

The vessel density effects of OSA have been examined by some authors through OCT angiography (OCT-A) and include reduced vessel density at the retinal capillary plexus [18,23]. In the short term, vasoconstriction of retinal vessels could lead to thinning of the more vascularized retinal layers. Some authors also propose that in the long term, this vasoconstriction could compromise the supply of nutrients to the inner retina and optic nerve head [11], thus increasing the risk of glaucoma in persons with OSA [24,25]. Glaucoma and OSA share the feature of vascular regulation abnormalities produced as the consequence of an imbalance in vasoactive substances. As normal-tension glaucoma is characterized by optic nerve damage in the presence of IOP values < 21 mmHg, its physiopathology could be the outcome of vascular anomalies [26]. Lin et al. [27] observed a prevalence of normotensive glaucoma of 7.1% among subjects with moderate to severe OSA. The prevalence detected here was also 7.1% (2/28) compared to a reported prevalence in the general population of 2% [28]. Therapy with CPAP increases the cross-sectional area of the upper airway through the application of positive pressure, avoiding respiratory collapse and allowing for continuous air flow during the respiratory cycle. This form of treatment thus eliminates the hypoxia caused by apnea and has been shown to reduce the risk of vascular events in subjects with OSA [20], and could also improve the vascular supply to the retina and optic disk. These benefits of treatment may explain the preserved visual function of our study participants over the 12 months of follow up. In effect, Lin et al. [29] noted an improved visual field in OSA patients receiving 3 months of CPAP and related this to the improved neurocognitive behavior associated with CPAP treatment that results from diminished day-time drowsiness [30].

At the structural level, retinal thickness remained stable after 3 and 12 months of starting CPAP therapy. We even observed the thickening of some of the layers examined. These changes were more significant for pRNFL and total retinal thickness. Because of anatomical vessel distribution at the retinal level, OCT segmentations performed at the level of the pRNFL and total retina as these layers have a greater probability of harboring vascular structures. Conversely, OCT cross-sections of the RGCL and IPL layers are narrower and less vascularized such that they have a smaller vessel proportion throughout their thickness. The outer retina is an avascular layer. Yu et al. [31] found that retinal thickness was strongly related to vascular perfusion, especially at the level of the pRNFL. Precisely at this level, we detected correlation with T90, mostly negative correlation with the thickness of the T subfield at the three time points. However, pRNFL SN subfield thickness was positively correlated with T90 after 12 months of CPAP. This discrepancy could be due to the different distribution of vessels at the peripapillary level. Abdullayev et al. [32] detected no significant changes in pRNFL and RGCL when they examined the effects of CPAP in a study conducted in 59 subjects with OSA, 28 of whom were treated with CPAP and 31 not receiving CPAP. However, the authors of this article did not consider adherence to treatment. Further, the OCT instrument used was a Cirrus HD-OCT. Another difference was that participants were individuals with mild, moderate, and severe OSA so their study population was more heterogeneous than ours.

Lin et al. described thickening of the pRNFL and total retina in a group of 32 subjects with OSA receiving 3 months of CPAP treatment after confirming their correct adherence to treatment [29]. We were able to confirm these findings, observing increased pRNFL and TR thickness in 28 OSA subjects showing good compliance with CPAP treatment [16]. The most repeated pattern observed here in pRNFL and TR thickness was a peak produced at 3 months followed by a moderate decrease at 12 months, but always featuring a greater thickness than that observed just before the start of CPAP. The human retina naturally loses thickness each year. Accordingly, a retina showing an average pRNFL thickness of 100 µm (97.98, 99.51, and 98.96 µm at baseline, 3, and 12 months, respectively, in the present study) will show a thickness loss of 0.55 µm/year [33]. In our study, the thickness loss in the 9 months between the first and second follow-up sessions was precisely 0.55 µm. Hence, the decrease in thickness observed between the two visits could be physiological. In a recent study by our group [16] and in line with the findings of others [10], we observed that the TIM subfield shows a greater susceptibility to thickening in subjects not undergoing CPAP, so we attributed this finding to inflammation. In the present study, we noted a greater pRNFL and TR thickness in the TIM subfield despite the use of CPAP. At the level of the layers RGCL and IPL, we observed isolated thickening in 1 and 2 subfields, respectively, after initiating CPAP therapy. These layers show a lesser vascular component.

The PL remained stable throughout the 12 months of follow up. In a prior study, we detected PL thickening in 6/9 subfields in subjects with OSA not treated with CPAP [16]. The low O_2_ levels associated with OSA give rise to the increased expression of vascular endothelial growth factor (VEGF) [34,35], causing destruction of the vascular endothelium and increased permeability from the choroid to the outer retina. Schaal et al. [36] observed a functional and structural improvement in patients with age-related macular degeneration receiving CPAP. We propose this therapy helps reduce permeability from the choroid to the outer retina as VEGF levels improve. Further, the photoreceptor layer is the tissue showing the greater metabolism of our organism [37]. Thus, in conditions of hypoxia, as occurs in OSA, we might expect to observe cell damage and inflammation, and as CPAP enhances O_2_ levels, we would expect to find the reduced inflammation and thickness of this layer.

At the choroid level, we observed positive correlation between choroidal thickness and AHI in the nasal subfield at the three study time points. This determined that subjects with more severe OSA showed a thicker choroid. We also observed a thickness decrease in all the subfields examined after starting with CPAP, which was significant in the temporal subfield 3 and 12 months after initiating treatment. In another study, we related choroid thinning in OSA subjects after 3 months of CPAP therapy [16] to a drop in intracranial pressure and improved hemodynamic and respiratory variables attributed to the use of CPAP [38]. However, this could be the consequence of an improvement in the autoregulation capacity of the choroid as endothelial damage improves [20]. A greater increase in choroid flow than expected has been shown in patients with glaucoma as a response to increased blood pressure produced during exercise [12]. The authors related this finding to choroid autoregulation abnormalities.

As CPAP therapy continues, systemic and respiratory disorders improve and the retinal and choroidal situation normalizes. We observed this here in that after the use of CPAP: (1) an increase was produced in retinal thickness, especially in regions with a greater vascular presence such as the pRNFL and TR layers which encompass all the vessels of the retina, superficial vascular plexus (greater caliber vessels), and deep vascular plexus (lesser caliber capillaries; and (2) hemodynamic variables improved at the systemic level, reducing intracranial pressure and thus supplying less blood to the choroid, promoting choroidal thinning.

As limitations, we should mention that we did not measure several factors such as CO_2_, VEGF, endothelin, nitric oxide, or molecules related to inflammation and oxidative stress [20,39] which could be correlated with some of the results observed here. We feel a complete year of follow up will help us understand the changes produced in the retina and choroid in OSA subjects receiving CPAP, yet a longer follow-up duration would be useful. It would also be interesting to include a control group of subjects with OSA not receiving CPAP treatment to distinguish between effects secondary to treatment and those secondary to OSA.

OSA is a chronic disease whose effects appear gradually over time. Most authors would argue that there is a need for long-term studies with a standardized rigorous methodology to improve our understanding of the real impacts of this disorder. As far as we know, this is the first study to monitor retinal and choroidal effects in OSA subjects under CPAP therapy for 12 months. The mechanical harmful IOP-increasing effect of CPAP was offset by an appreciable improvement in the vascular bed at the level of the retina and optic nerve. In addition, no detrimental effects were observed in the visual field, and there was no retinal thickness loss over the study year. Thus, from a clinical perspective, we should highlight that CPAP could protect against glaucoma in individuals with OSA. Similarly, we detected thinning of the choroid and preserved PL thickness, also suggesting that CPAP could protect against outer retinal disease in OSA.

## Figures and Tables

**Table 1:** Baseline characteristics of the patients with obstructive sleep apnea included in this study.

	Patients No. (% or ± SD) *n* = 28
Sex (men)	26 (92.9)
Age (years)	57 (±9)
BMI (Kg/m^2^)	32.1 (±4.8)
Glaucoma (%)	2 (7.14)
AMI (%)	1 (3.6)
Smoker (%)	4 (14.3)
AHT (%)	12 (42.9)
Hyperlipidemia (%)	11 (39.3)
DMII (%)	7 (25.0)
Hyperuricemia (%)	4 (14.3)
CKD (%)	1 (3.6)
Anemia (%)	1 (3.6)
Psoriasis (%)	1 (3.6)
Hypothyroidism (%)	1 (3.6)
Arrhythmia (%)	1 (3.6)
Severe OSA (%)	27 (96.4)
AHI (events/h)	61.8 (±21.5)
CPAP use (h)	5.09 (±1.83)
ODI (events/h)	66.8 (±21.6)
T90 (%)	13.0 (±12.2)
SpO_2_ mean (%)	91.2 (±3.5)
SpO_2_ minimum (%)	77.0 (±10.7)

Categorical variables expressed as percentages. Continuous variables expressed as mean (±SD, standard deviation). BMI = body mass index, AMI = acute myocardial infarction, AHT = arterial hypertension, DMII = diabetes mellitus type II, CKD = chronic kidney disease, OSA = obstructive sleep apnea, AHI = apnea hypopnea index, CPAP = continuous positive airway pressure, ODI = oxygen desaturation index, SpO_2_ = peripheral oxygen saturation, T90 = percentage sleep time spent at SpO_2_ ≤ 90%. Reference [16].

**Table 2:** Variables recorded at baseline and at 3 and 12 months of follow up in patients with obstructive sleep apnea undergoing continuous positive airway pressure treatment.

	Baseline	3 Months	12 Months	P* Raw	P** Adjusted (1) (2) (3)
IOP (mmHg)	14.65 (±3.27)	14.13 (±2.41)	15.27 (±2.93)	**0.039**	0.241 0.231 **0.001**
LogMAR	−0.04 (±0.08)	−0.06 (±0.09)	−0.07 (±0.08)	0.121	NA
VFI (%)	98.13 (±2.21)	97.81 (±2.44)	98.38 (±1.76)	0.499	NA
VF MD (decibels)	−2.20 (±1.24)	−2.16 (±1.23)	−2.10 (±1.04)	0.472	NA

Data expressed as the mean (±standard deviation). P* raw: significance level obtained through the Friedman test. P** adjusted: significance level adjusted through the Wilcoxon test as a post-hoc test for multiple comparisons. (1) Baseline vs. 3 months. (2) Baseline vs. 12 months. (3) 3 months vs. 12 months. NA: Post-hoc test not applicable for a P* greater than 0.1. Values in bold indicate *p* < 0.05. IOP = intraocular pressure, LogMAR = logarithm of the minimum angle of resolution, VFI = visual field index, VF MD = visual field mean deviation.

**Table 3:** Retinal nerve fiber layer thickness at the peripapillary level recorded at baseline and at 3 and 12 months of follow up in patients with obstructive sleep apnea undergoing continuous positive airway pressure treatment.

	Baseline	3 Months	12 Months	P* Raw	P** Adjusted (1) (2) (3)
RNFL_G	97.98 (±9.12)	99.51 (±9.55)	98.96 (±9.02)	**<0.001**	**<0.001** **0.012** 0.535
RNFL_T	72.94 (±12.67)	74.24 (±13.60)	73.69 (±12.61)	**0.046**	**0.003** 0.364 0.999
RNFL_ST	135.80 (±21.44)	136.04 (±21.83)	136.57 (±21.26)	0.581	NA
RNFL_SN	111.94 (±24.92)	114.37 (±25.12)	113.06 (±24.36)	**0.014**	**0.004** 0.170 0.215
RNFL_N	72.43 (±11.45)	74.43 (±11.63)	73.24 (±10.81)	**0.001**	**0.001** 0.392 0.137
RNFL_IN	101.94 (±20.31)	103.82 (±20.38)	103.94 (±20.25)	**0.030**	**0.001** 0.050 0.237
RNFL_IT	143.49 (±18.88)	144.82 (±20.31)	143.61 (±18.25)	0.135	NA

Data expressed as the mean (±standard deviation). P* raw: significance level obtained by repeated measures ANOVA or Friedman test (according to normality of data distribution). P** adjusted: adjusted significance level obtained through post-hoc tests for multiple comparisons: Bonferroni or Wilcoxon (according to normality of data distribution). (1) Baseline vs. 3 months. (2) Baseline vs. 12 months. (3) 3 months vs. 12 months. NA: Post-hoc test not applicable for P* greater than 0.1. Values in bold indicate *p* < 0.05. RNFL_G = retinal nerve fiber layer global, T = temporal, ST = superotemporal, SN = superonasal, N = nasal, IN = inferonasal, IT = inferotemporal.

**Table 4:** Thickness measurements made at the macular level at baseline and at 3 and 12 months of follow up in patients with obstructive sleep apnea undergoing continuous positive airway pressure treatment.

	Baseline	3 Months	12 Months	P* Raw	P** Adjusted (1) (2) (3)
TR					
F	284.82 (±20.93)	286.61 (±21.51)	286.37 (±20.96)	**0.035**	**0.010** 0.134 0.999
IIM	342.90 (±17.56)	345.10 (±18.46)	344.75 (±18.05)	**<0.001**	**<0.001** **<0.001** 0.999
IOM	289.10 (±16.79)	290.55 (±17.42)	290.06 (±17.37)	**0.003**	**0.007** 0.081 0.659
TIM	332.82 (±16.20)	333.53 (±20.05)	335.29 (±16.09)	**0.091**	0.999 **<0.001** 0.496
TOM	285.57 (±21.00)	286.94 (±17.27)	284.80 (±15.97)	**0.003**	**<0.001** 0.108 0.120
SIM	346.51 (±17.68)	348.25 (±18.53)	347.47 (±18.08)	**0.001**	**0.001** 0.139 0.245
SOM	297.71 (±17.20)	299.04 (±18.53)	298.06 (±17.41)	**0.018**	0.057 0.999 0.079
NIM	348.31 (±16.48)	350.92 (±18.33)	350.98 (±17.66)	**0.016**	0.056 0.062 0.999
NOM	316.53 (±20.07)	317.55 (±18.80)	315.94 (±18.75)	0.199	NA
RGCL					
F	18.85 (±6.22)	18.60 (±6.04)	18.40 (±6.05)	0.232	NA
IIM	53.35 (±5.35)	53.54 (±5.99)	53.40 (±5.77)	0.787	NA
IOM	33.44 (±4.43)	33.31 (±4.31)	33.58 (±3.89)	0.107	NA
TIM	49.48 (±5.91)	49.29 (±6.46)	49.52 (±6.04)	0.650	NA
TOM	36.17 (±4,74)	36.23 (±5.07)	36.06 (±4.75)	0.796	NA
SIM	53.79 (±5.26)	54.12 (±5.61)	54.63 (±5.86)	**<0.001**	0.312 **<0.001** **0.048**
SOM	34.98 (±3.98)	35.10 (±4.02)	35.15 (±4.07)	0.623	NA
NIM	52.75 (±6.12)	52.40 (±6.87)	52.71 (±6.61)	0.712	NA
NOM	38.13 (±4.58)	38.46 (±5.09)	38.62 (±5.41)	0.173	NA
IPL					
F	23.77 (±4.36)	23.79 (±4.46)	23.75 (±4.15)	0.988	NA
IIM	41.92 (±4.06)	42.29 (±4.04)	42.37 (±4.30)	0.161	NA
IOM	27.02 (±3.24)	27.23 (±3.40)	27.04 (±3.15)	0.375	NA
TIM	42.40 (±3.99)	43.25 (±4.03)	43.10 (±4.04)	**0.004**	**0.002** 0.056 0.999
TOM	32.67 (±3.44)	33.02 (±3.34)	32.81 (±3.38)	0.107	NA
SIM	42.48 (±3.76)	42.85 (±3.56)	42.52 (±4.27)	0.261	NA
SOM	28.58 (±3.24)	28.77 (±3.42)	28.77 (±3.31)	0.281	NA
NIM	43.46 (±3.88)	43.77 (±4.00)	44.02 (±3.81)	**0.079**	0.061 **0.046** 0.999
NOM	29.33 (±3.27)	29.54 (±3.23)	29.31 (±3.31)	0.226	NA
PL					
F	87.85 (±4.33)	87.71 (±4.92)	87.50 (±3.80)	0.650	NA
IIM	79.08 (±2.70)	79.52 (±3.05)	78.60 (±4.90)	0.146	NA
IOM	76.81 (±2.23)	77.08 (±2.19)	75.92 (±5.09)	0.166	NA
TIM	80.08 (±2.91)	80.33 (±2.87)	79.69 (±3.03)	0.105	NA
TOM	77.15 (±2.38)	77.37 (±2.43)	76.60 (±4.12)	0.466	NA
SIM	80.25 (±2.81)	80.17 (±2.86)	79.88 (±2.72)	0.847	NA
SOM	78.52 (±2.81)	78.65 (±2.56)	78.06 (±3.23)	0.178	NA
NIM	80.98 (±3.03)	81.62 (±3.19)	80.98 (±3.37)	0.130	NA
NOM	77.94 (±2.51)	78.31 (±2.38)	77.65 (±4.41)	0.318	NA

Data expressed as the mean (±standard deviation). P* raw: significance level obtained by repeated measures ANOVA or Friedman test (according to normality of data distribution). P** adjusted: adjusted significance level obtained through post-hoc tests for multiple comparisons: Bonferroni or Wilcoxon (according to normality of data distribution). (1) Baseline vs. 3 months. (2) Baseline vs. 12 months. (3) 3 months vs. 12 months. NA: Post-hoc test not applicable for P* greater than 0.1. Values in bold indicate *p* < 0.05. TR = total retinal, F = fovea, IIM = inferior inner macula, IOM = inferior outer macula, TIM = temporal inner macula, TOM = temporal outer macula, SIM = superior inner macula, SOM = superior outer macula, NIM = nasal inner macula, NOM = nasal outer macula, RGCL = retinal ganglion cell layer, IPL = inner plexiform layer, PL = photoreceptor layer.

**Table 5:** Choroidal thickness recorded at baseline and at 3 and 12 months of follow up in patients with obstructive sleep apnea undergoing continuous positive airway pressure treatment.

	Baseline	3 Months	12 Months	P* Raw	P** Adjusted (1) (2) (3)
Fovea	268.84 (±61.66)	257.68 (±61.16)	262.36 (±59.63)	0.079	0.116 0.456 0.999
Temporal	259.75 (±65.06)	247.52 (±59.33)	250.73 (±60.19)	**0.007**	**0.014** **0.038** 0.999
Nasal	249.70 (±66.64)	246.52 (±66.57)	244.86 (±64.61)	0.517	NA

Data expressed as the mean (±standard deviation). P* raw: significance level obtained by repeated measures ANOVA or Friedman test (according to normality of data distribution). P** adjusted: adjusted significance level obtained through post-hoc tests for multiple comparisons with Bonferroni correction. (1) Baseline vs. 3 months. (2) Baseline vs. 12 months. (3) 3 months vs. 12 months. NA: Post-hoc test not applicable for P* greater than 0.1. Values in bold indicate *p* < 0.05.

## Data Availability

The datasets used and/or analyzed during the current study are available from the corresponding authors on reasonable request.

## Abbreviations

AHI	apnea hypopnea index
CPAP	continuous positive airway pressure
F	fovea
G	global
ICC	intraclass correlation coefficient
IIM	inferior inner macula
IN	inferonasal
IOM	inferior outer macula
IOP	intraocular pressure
IPL	inner plexiform layer
IT	inferotemporal
N	nasal
NIM	nasal inner macula
NOM	nasal outer macula
OSA	obstructive sleep apnea
PL	photoreceptor layer
RGCL	retinal ganglion cell layer
RNFL	retinal nerve fiber layer
pRNFL	peripapillary retinal nerve fiber layer
SAP	standard automated perimetry
SIM	superior inner macula
SN	superonasal
SOM	superior outer macula
SpO_2_	peripheral oxygen saturation
ST	superotemporal
T	temporal
TIM	temporal inner macula
TOM	temporal outer macula
TR	total retinal
T90	percentage sleep time spent at SpO_2_ ≤ 90%
VEGF	vascular endothelial growth factor
IOP	intraocular pressure
SD-OCT	spectral domain ocular coherence tomography
OCT-A	ocular coherence tomography angiography

## References

[1] Heinzer R., Vat S., Marques-Vidal P., Marti-Soler H., Andries D., Tobback N., Mooser V., Preisig M., Malhotra A., Waeber G. (2015). Prevalence of sleep-disordered breathing in the general population: The HypnoLaus study. Lancet Respir. Med..

[2] Young T., Peppard P.E., Gottlieb D.J. (2002). Epidemiology of obstructive sleep apnea: A population health perspective. Am. J. Respir. Crit. Care Med..

[3] Baratta F., Pastori D., Fabiani M., Fabiani V., Ceci F., Lillo R., Lolli V., Brunori M., Pannitteri G., Cravotto E. (2018). Severity of OSAS, CPAP and cardiovascular events: A follow-up study. Eur. J. Clin. Investig..

[4] Levy P., Kohler M., McNicholas W.T., Barbe F., McEvoy R.D., Somers V.K., Lavie L., Pepin J.L. (2015). Obstructive sleep apnoea syndrome. Nat. Rev. Dis. Prim..

[5] Wu C.Y., Riangwiwat T., Rattanawong P., Nesmith B.L.W., Deobhakta A. (2018). Association of obstructive sleep apnea with central serous chorioretinopathy and choroidal thickness: A Systematic Review and Meta-Analysis. Retina.

[6] Lin P.W., Friedman M., Lin H.C., Chang H.W., Pulver T.M., Chin C.H. (2011). Decreased retinal nerve fiber layer thickness in patients with obstructive sleep apnea/hypopnea syndrome. Graefe’s Arch. Clin. Exp. Ophthalmol..

[7] Zengin M.O., Tuncer I., Karahan E. (2014). Retinal nerve fiber layer thickness changes in obstructive sleep apnea syndrome: One year follow-up results. Int. J. Ophthalmol..

[8] Shiba T., Takahashi M., Sato Y., Onoda Y., Hori Y., Sugiyama T., Bujo H., Maeno T. (2014). Relationship between severity of obstructive sleep apnea syndrome and retinal nerve fiber layer thickness. Am. J. Ophthalmol..

[9] Hashim S.P., Al Mansouri F.A., Farouk M., Al Hashemi A.A., Singh R. (2014). Prevalence of glaucoma in patients with moderate to severe obstructive sleep apnea: Ocular morbidity and outcomes in a 3 year follow-up study. Eye.

[10] Casas P., Ascaso F.J., Vicente E., Tejero-Garces G., Adiego M.I., Cristobal J.A. (2013). Retinal and optic nerve evaluation by optical coherence tomography in adults with obstructive sleep apnea-hypopnea syndrome (OSAHS). Graefe’s Arch. Clin. Exp. Ophthalmol..

[11] Kur J., Newman E.A., Chan-Ling T. (2012). Cellular and physiological mechanisms underlying blood flow regulation in the retina and choroid in health and disease. Prog. Retin. Eye Res..

[12] Portmann N., Gugleta K., Kochkorov A., Polunina A., Flammer J., Orgul S. (2011). Choroidal blood flow response to isometric exercise in glaucoma patients and patients with ocular hypertension. Investig. Ophthalmol. Vis. Sci..

[13] Papst N., Demant E., Niemeyer G. (1982). Changes in pO2 induce retinal autoregulation in vitro. Graefe’s Arch. Clin. Exp. Ophthalmol..

[14] Jelic S., Padeletti M., Kawut S.M., Higgins C., Canfield S.M., Onat D., Colombo P.C., Basner R.C., Factor P., LeJemtel T.H. (2008). Inflammation, oxidative stress, and repair capacity of the vascular endothelium in obstructive sleep apnea. Circulation.

[15] Chang M., Yoo C., Kim S.W., Kim Y.Y. (2011). Retinal vessel diameter, retinal nerve fiber layer thickness, and intraocular pressure in korean patients with normal-tension glaucoma. Am. J. Ophthalmol..

[16] Naranjo-Bonilla P., Munoz-Villanueva M.C., Gimenez-Gomez R., Jurado-Gamez B. (2021). Retinal and choroidal thickness measurements in obstructive sleep apnea: Impacts of continuous positive airway pressure treatment. Graefe’s Arch. Clin. Exp. Ophthalmol..

[17] Zheng Y., Cheung N., Aung T., Mitchell P., He M., Wong T.Y. (2009). Relationship of retinal vascular caliber with retinal nerve fiber layer thickness: The singapore malay eye study. Investig. Ophthalmol. Vis. Sci..

[18] Ucak T., Unver E. (2020). Alterations in Parafoveal and Optic Disc Vessel Densities in Patients with Obstructive Sleep Apnea Syndrome. J. Ophthalmol..

[19] Medeiros F.A., Lisboa R., Weinreb R.N., Liebmann J.M., Girkin C., Zangwill L.M. (2013). Retinal ganglion cell count estimates associated with early development of visual field defects in glaucoma. Ophthalmology.

[20] Jurado-Gamez B., Fernandez-Marin M.C., Gomez-Chaparro J.L., Munoz-Cabrera L., Lopez-Barea J., Perez-Jimenez F., Lopez-Miranda J. (2011). Relationship of oxidative stress and endothelial dysfunction in sleep apnoea. Eur. Respir. J..

[21] Lloberes P., Duran-Cantolla J., Martinez-Garcia M.A., Marin J.M., Ferrer A., Corral J., Masa J.F., Parra O., Alonso-Alvarez M.L., Teran-Santos J. (2011). Diagnosis and treatment of sleep apnea-hypopnea syndrome. Spanish Society of Pulmonology and Thoracic Surgery. Arch. Bronconeumol..

[22] Jurado-Gamez B., Bardwell W.A., Cordova-Pacheco L.J., Garcia-Amores M., Feu-Collado N., Buela-Casal G. (2015). A basic intervention improves CPAP adherence in sleep apnoea patients: A controlled trial. Sleep Breath..

[23] Yu J., Xiao K., Huang J., Sun X., Jiang C. (2017). Reduced Retinal Vessel Density in Obstructive Sleep Apnea Syndrome Patients: An Optical Coherence Tomography Angiography Study. Investig. Ophthalmol. Vis. Sci..

[24] Wang J.S., Xie H.T., Jia Y., Zhang M.C. (2016). Retinal nerve fiber layer thickness changes in obstructive sleep apnea syndrome: A systematic review and Meta-analysis. Int. J. Ophthalmol..

[25] Shi Y., Liu P., Guan J., Lu Y., Su K. (2015). Association between glaucoma and obstructive sleep apnea syndrome: A meta-analysis and systematic review. PLoS ONE.

[26] Kivela T., Hietanen J., Uusitalo M. (1997). Autopsy analysis of clinically unilateral exfoliation syndrome. Investig. Ophthalmol. Vis. Sci..

[27] Lin P.W., Friedman M., Lin H.C., Chang H.W., Wilson M., Lin M.C. (2011). Normal tension glaucoma in patients with obstructive sleep apnea/hypopnea syndrome. J. Glaucoma.

[28] Mojon D.S., Hess C.W., Goldblum D., Fleischhauer J., Koerner F., Bassetti C., Mathis J. (1999). High prevalence of glaucoma in patients with sleep apnea syndrome. Ophthalmology.

[29] Lin P.W., Lin H.C., Friedman M., Chang H.W., Salapatas A.M., Lin M.C., Chen Y.C. (2020). Effects of CPAP for patients with OSA on visual sensitivity and retinal thickness. Sleep Med..

[30] Antic N.A., Catcheside P., Buchan C., Hensley M., Naughton M.T., Rowland S., Williamson B., Windler S., McEvoy R.D. (2011). The effect of CPAP in normalizing daytime sleepiness, quality of life, and neurocognitive function in patients with moderate to severe OSA. Sleep.

[31] Yu J., Gu R., Zong Y., Xu H., Wang X., Sun X., Jiang C., Xie B., Jia Y., Huang D. (2016). Relationship Between Retinal Perfusion and Retinal Thickness in Healthy Subjects: An Optical Coherence Tomography Angiography Study. Investig. Ophthalmol. Vis. Sci..

[32] Abdullayev A., Tekeli O., Yanik O., Acican T., Gulbay B. (2019). Investigation of the Presence of Glaucoma in Patients with Obstructive Sleep Apnea Syndrome Using and Not Using Continuous Positive Airway Pressure Treatment. Turk. J. Ophthalmol..

[33] Leung C.K., Ye C., Weinreb R.N., Cheung C.Y., Qiu Q., Liu S., Xu G., Lam D.S. (2010). Retinal nerve fiber layer imaging with spectral-domain optical coherence tomography a study on diagnostic agreement with Heidelberg Retinal Tomograph. Ophthalmology.

[34] Dioum E.M., Chen R., Alexander M.S., Zhang Q., Hogg R.T., Gerard R.D., Garcia J.A. (2009). Regulation of hypoxia-inducible factor 2alpha signaling by the stress-responsive deacetylase sirtuin 1. Science.

[35] Liu J., Narasimhan P., Yu F., Chan P.H. (2005). Neuroprotection by hypoxic preconditioning involves oxidative stress-mediated expression of hypoxia-inducible factor and erythropoietin. Stroke.

[36] Schaal S., Sherman M.P., Nesmith B., Barak Y. (2016). Untreated Obstructive Sleep Apnea Hinders Response to Bevacizumab in Age-Related Macular Degeneration. Retina.

[37] Sivaprasad S., Arden G. (2016). Spare the rods and spoil the retina: Revisited. Eye.

[38] Lee A.G., Golnik K., Kardon R., Wall M., Eggenberger E., Yedavally S. (2002). Sleep apnea and intracranial hypertension in men. Ophthalmology.

[39] Jurado-Gamez B., Bujalance Cabrera C., Caballero Ballesteros L., Marin Hinojosa C., Munoz Cabrera L., Perez-Jimenez F., Lopez-Miranda J. (2012). Association of cellular adhesion molecules and oxidative stress with endothelial function in obstructive sleep apnea. Intern. Med..

